# The Impact of Long COVID on Employment and Well-Being: A Qualitative Study of Patient Perspectives

**DOI:** 10.1007/s11606-024-09062-5

**Published:** 2024-10-08

**Authors:** Sarah R. MacEwan, Saurabh Rahurkar, Willi L. Tarver, Leanna Perez Eiterman, Halia Melnyk, Ramona G. Olvera, Jennifer L. Eramo, Lauren Teuschler, Alice A. Gaughan, Laura J. Rush, Stacy Stanwick, Susan Bowman Burpee, Erin McConnell, Andrew Schamess, Ann Scheck McAlearney

**Affiliations:** 1 Division of General Internal Medicine, College of Medicine, The Ohio State University, Columbus, OH USA; 2 Center for the Advancement of Team Science, Analytics, and Systems Thinking in Health Services and Implementation Science Research (CATALYST), College of Medicine, The Ohio State University, Columbus, OH USA; 3 Department of Biomedical Informatics, College of Medicine, The Ohio State University, Columbus, OH USA; 4 Division of Cancer Prevention and Control, The Ohio State University, Columbus, OH USA; 5 Department of Physical Medicine and Rehabilitation, College of Medicine, The Ohio State University, Columbus, OH USA; 6 Department of Family and Community Medicine, College of Medicine, The Ohio State University, Columbus, OH USA

**Keywords:** post-acute COVID-19 syndrome, chronic disease, work, disability, Long COVID

## Abstract

**Background:**

Exploring the experiences of Long COVID patients who face challenges with employment may inform improvements in how healthcare systems can provide holistic care for this patient population.

**Objective:**

Understand perspectives about the impact of Long COVID on employment and well-being among patients seeking healthcare for Long COVID.

**Design:**

Qualitative study involving one-on-one interviews.

**Participants:**

Eligible participants were 18 years of age or older, spoke English, self-reported as doing well in daily life before having COVID-19, and reported that COVID-19 was still having a significant impact on their life three or more months following an acute infection.

**Approach:**

Participants were recruited from a post-COVID recovery clinic at an academic medical center. Interviews were conducted from August to September 2022.

**Key Results:**

Among all participants (*N* = 21), most described that they were not able to work at a capacity equivalent to their norm pre-COVID-19. For those who continued working after their COVID-19 infection, the effort and energy required for work left little capacity to participate in other life activities and made it difficult to attend recommended healthcare appointments. Participants reported financial impacts of changes in employment including loss of income and changes in insurance, which were compounded by high healthcare costs. Changes in employment resulted in emotional repercussions including feelings of loss of self-identity and fear of judgement at work. Participants discussed issues surrounding access to strategies to address challenges posed by the impact of Long COVID on employment, including strategies learned from healthcare providers to cope with Long COVID symptoms at work and efforts to seek disability benefits or workplace accommodations.

**Conclusions:**

Patients with Long COVID may face significant challenges due to changes in their ability to work. Healthcare providers can support these patients by connecting them to financial assistance resources, facilitating appropriate mental health treatment, and expediting workplace accommodation requests.

**Supplementary Information:**

The online version contains supplementary material available at 10.1007/s11606-024-09062-5.

## INTRODUCTION

Long COVID is a chronic condition in which symptoms after COVID-19 infection persist for at least three months.^1^ Some individuals with Long COVID have limitations in their activities and symptoms severe enough to cause disability, both of which can impact employment. Among a sample of individuals with Long COVID, approximately one-quarter reported significant activity limitations,^2^ and nearly two-thirds reported a disability.^3^ Individuals with Long COVID have been shown to have a lower likelihood of full-time employment and a higher likelihood of unemployment compared to individuals without Long COVID.^4^ A greater degree of Long COVID symptom burden (e.g., cognitive symptoms) has also been shown to be associated with lower likelihood of working full-time.^5^ Twenty-two percent of individuals with Long COVID have reported they were unable to work 6 months after initial infection, 45% required a reduced work schedule,^6^ and 18% were unable to return to work a year after COVID-19 infection.^7^ Due to this reduced capacity to work, it is estimated that Long COVID resulted in a minimum of $170 billion in lost wages in the USA in 2022.^8^

While disruption of employment due to Long COVID and its societal implications are being recognized, it is also important to acknowledge the lived experiences and unmet needs of individuals coping with changes in employment caused by this chronic condition. Prior studies have described experiences of individuals with Long COVID as they navigate employer and coworker relationships, workplace accommodations, and loss of personal identity related to changes in their employment.^9–16^ However, there remains a gap in understanding the perspectives of patients receiving healthcare for Long COVID regarding these issues. These perspectives are needed to help guide healthcare systems and providers in how they can help Long COVID patients facing challenges with employment. The goal of this study is therefore to understand perspectives about the impact of Long COVID on employment, and the repercussions of this impact on well-being, among patients receiving healthcare for Long COVID. Our findings can help inform efforts to improve support in healthcare settings for Long COVID patients experiencing reduced capacity to work.

## METHODS

### Study Design and Research Team

This study utilized a qualitative description approach involving one-on-one interviews^17–19^ to improve our understanding of the perspectives of Long COVID patients. Our research team included health services researchers as well as clinicians who provide healthcare to patients with Long COVID within the post-COVID recovery clinic at an academic medical center (AMC).

### Study Population

Interviews were conducted from August to September 2022 with English-speaking adults (age ≥ 18 years) who were receiving care at a post-COVID recovery clinic associated with the AMC. Purposeful sampling was used to identify eligible participants who self-reported doing well in daily life before having COVID-19 and for whom COVID-19 was still having a significant impact on their life three or more months following their acute infection. There were no exclusion criteria for study participation.

### Data Collection

Potential participants were approached by clinic staff who explained the study and inquired about interest in participation. Contact information of interested patients was provided to the research team who then reached out by email to schedule an interview. Five members of the research team, including female and male master’s- and PhD-trained health services researchers, conducted interviews by phone or videoconference using a semi-structured interview guide. Patients did not know their interviewer prior to their interview. Participants provided verbal informed consent. All interviews were audio recorded. Patients received a $25 electronic gift card in appreciation for their time. The Ohio State University’s Institutional Review Board approved this study (study ID: 2020B0288).

### Data Analysis

Interview audio recordings were transcribed and analyzed by deductive and inductive thematic analysis.^20^ A preliminary coding dictionary was created based on topics included in the semi-structured interview guide. Four of the research team members who conducted the interviews independently coded one transcript using this preliminary coding dictionary and met to compare application of the codes. Group discussion informed refinement of code definitions and the addition of emergent codes. The team divided remaining transcripts among themselves and applied codes across all transcripts, meeting weekly to address questions about application of codes that were resolved by team consensus.

Within the revised coding dictionary, several codes were relevant to patient perspectives about the impact of Long COVID on employment. These codes captured parts of the interview transcripts that described participants’ perspectives about the impact of Long COVID on employment and school, financial concerns, and coping strategies, as well as experiences of loss and stigma. Transcript excerpts that were identified with these codes were collectively reviewed and emergent themes and subthemes were identified that encompassed participants’ perspectives about the impact of Long COVID on employment. ATLAS.ti qualitative analysis software (version 23.2.1; ATLAS.ti Scientific Software Development GmbH) was used to facilitate this process.

## RESULTS

Interviews were conducted with 21 patients (Table 1) and lasted an average of 49.5 min. Participants had a mean age of 47.6 years (range: 19–68). The majority (76%) were female. The plurality lived in a suburban area and were first infected with COVID-19 in 2020.

**Table 1:** Characteristics of Patient Participants with Long COVID

Characteristic	*n* (%) *N* = 21
Age (years)	
18–44	7 (33)
45–54	7 (33)
55–64	5 (24)
65 +	2 (10)
Sex	
Female	16 (76)
Male	5 (24)
Geographic area	
Urban	7 (33)
Suburban	10 (48)
Rural	4 (19)
Year of first COVID-19 infection	
2020	10 (48)
2021	6 (28)
2022	5 (24)

Participants described their experience with Long COVID and its impact on their employment. For some, this impact was transient, causing temporary changes that had since resolved, allowing them to return to what was at or near the norm of their pre-COVID-19 employment. For others, the impact of Long COVID on their ability to work was long lasting and unabated, with some participants describing changes in employment, challenges in continuing to work, or the inability to return to work. Participants’ perspectives about the impact of Long COVID on employment and well-being focused on four themes: (1) impact of Long COVID on the ability to work; (2) financial consequences; (3) emotional repercussions; and (4) access to strategies to address challenges posed by the impact of Long COVID on employment. Themes, subthemes, and representative quotations are presented in Table 2.

**Table 2:** Themes, Subthemes, and Representative Quotations About the Impact of Long COVID on Employment and Well-Being

Theme	Subtheme	Representative quotation
Impact of Long COVID on the ability to work	Interference of Long COVID symptoms with work responsibilities	I’m not currently working. Matter of fact, doctor highly recommended I look at filing for some type of disability or retraining into an office job or some other type of job … where I could be in a controlled climate and I’m not physically exerting myself. (P13)
	Impact of Long COVID on work-life balance	Literally for the first six months, all I did was work and go to bed. I was either sleeping or working. Yeah, there was nothing else–or doctor’s appointments. (P3)
Financial consequences from the impact of Long COVID on employment	Loss of income	I mean losing my job, it took me roughly four months to find another employer. And with that unemployment, it’s a tiny fraction of what I had made before. And so, it wasn’t just getting by, it was like, okay, now I have to sell some assets. Things that are very dear to me, I’m gonna have to let them go because I still have bills to pay. (P20)
	Changes in insurance	We took away my salary, our insurance. We have to pay through the marketplace now, and it’s very expensive. So, it’s definitely hurting us. I mean, we’re getting by, but we’re … Jesus, I don’t know. We’re probably going to have to tap into our retirement funds. It’s really impacted our life financially, medically, just everything. (P6)
Emotional repercussions from the impact of Long COVID on employment	Feelings of loss	As a young adult I’m kind of moving into the world of independence. And I’ve lost any semblance of an independence I had, not being able to have a job, not being able to work, not being able to even being at college. It’s difficult. (P10)
	Fear of exposure, judgement, stigma, and/or retaliation	That was what I asked the doctor to focus in on, was the brain fog like, help me get through some of this. Because I’ve been in hiding for over a year at my job and I can’t hide any longer. I have to either be productive or figure out how to move on. (P16)
Access to strategies to address challenges posed by the impact of Long COVID on employment	Coping strategies	For me, it’s very helpful to have visual prompts. … I have a little white board over here with days of the week, like the critical things, like a critical meeting I can’t miss or something, then I’ll write that on my little whiteboard. I keep a notebook where I write down to-dos or things that I need. Also, she [speech therapist] was talking to me about self-talk. So, sometimes when you’re doing something, it’s helpful if you can talk yourself through it and say okay, yes, I’m adding this to this list. Now, I’m going to send this email. (P1)
	Employer disability benefits and workplace accommodations	I asked her [doctor] because I read it in the newspaper somewhere about this Long COVID being classified as a disability and wondering when and if I might be able to collect disability benefits. (P4)

### Impact of Long COVID on the Ability to Work

We identified two subthemes around patients’ perspectives about how Long COVID impacted their ability to work: (1) interference of Long COVID symptoms with work responsibilities and (2) impact of Long COVID on work-life balance (see Appendix Table 1 for additional supporting quotations). Patients described experiencing a variety of Long COVID symptoms that impacted their ability to work. Brain fog, fatigue, weakness/lack of physical strength, and headaches/migraines were the symptoms mentioned most frequently. These Long COVID symptoms caused challenges in executing work tasks or limited an individual’s ability to perform work in an acceptable timeframe or volume. For some, Long COVID symptoms led to changes in employment status. Several participants described voluntarily leaving their jobs, which they felt they could no longer perform. Others described seeking alternative jobs that could better accommodate their symptoms. For some, these decisions were made based on recommendations from their healthcare team. Additionally, some described how the impact of their Long COVID symptoms on their ability to perform work tasks led to them being involuntarily terminated:

I was presenting … and I started stammering over words. … I politely explained that, ‘Hey, I’m sorry. I suffer from Long COVID, so it takes me a little bit here.’ And [they] didn’t care. … My credibility had been immediately diminished. (P20)

Long COVID also impacted individuals’ work-life balance. Many participants reported that work depleted their limited amount of energy or focus, leaving little opportunity for other activities in their life. Participants also commented on the challenges of balancing work with attending healthcare appointments due to time and energy constraints. For some participants, this contributed to their decision to change jobs. For others, this led to discontinuing healthcare services or activities recommended for their Long COVID treatment. One participant described this challenge:

I just realized that there was no way that I could work full-time and actually get to these medical appointments and, you know, also work on my degree, and do the laundry, and make sure the kids have food to eat. (P4)

### Financial Consequences from the Impact of Long COVID on Employment

Two subthemes were identified about the financial consequences from the impact of Long COVID on employment: (1) loss of income and (2) changes in insurance (see Appendix Table 2 for additional supporting quotations). Patients attributed loss of income caused by Long COVID to several factors: being unable to return to work, having to change jobs to lower paying positions, or having to limit work hours. For those who could return to work, several reported no longer being able to work overtime or pursue additional part-time work, which they had relied on to supplement their income prior to having COVID-19. One participant described having “just a decreased [budget] overall because I’m not doing contract work. I don’t, like, pick up extra hours at work any longer really. Some weeks I can, but it’s not sustainable like it was before.” (P2).

Changes in employment caused by Long COVID also precipitated changes in insurance that impacted individuals’ financial circumstances as well as their access to healthcare. In several instances, patients described changes in employment that necessitated they switch to more expensive insurance policies with less coverage. Some participants reported that these changes in insurance also led to increased expenses caused by the consequent reset for their deductibles and out-of-pocket maximums. One participant who was fired from their job due to their Long COVID symptoms described the impact of the resultant change in insurance:

It is not as good as the insurance I had from [previous employer]. It’s a higher deductible, higher out-of-pocket. So, now I’m on a payment plan … plus, I’m not maxed out on the out-of-pocket yet on this plan. (P3)

Decreased income and changes in insurance coincided with increased healthcare expenses, and some participants reported accumulating debt due to their Long COVID experiences.

### Emotional Repercussions from the Impact of Long COVID on Employment

Two subthemes were identified that described the emotional repercussions from the impact of Long COVID on employment: (1) feelings of loss and (2) fear of judgement, stigma, and/or retaliation (see Appendix Table 3 for additional supporting quotations). Patients described a feeling of loss of their identity due to changes in their ability to work. These feelings included their loss of identity as a provider for their family that was accompanied by feelings of guilt when describing barriers to earning a living commensurate to what they earned prior to contracting COVID-19. One participant described the impact of losing their identity as a provider for their family:

To not be able to provide, I think was the biggest blow. … It’s just part of who I am to provide for my family and be able to take care of things like that. And not being able to do that was really scary for me. (P3)

While some participants described employment environments in which they felt safe expressing their limitations related to Long COVID, other participants described fear of retaliation in their workplace. These feelings were reportedly fueled by negative experiences with employers that included challenges receiving work accommodations or resulted in termination. Some participants also expressed feelings of judgement or stigma from family and friends due to their diminished ability to work. One participant described the psychological impact they experienced after changing jobs:

It was scary, a lot of anxiety, not knowing if I would be able to perform. It’s a lot of emotional strain involved in it, still is. I have to be very guarded with what I say … I keep it entirely to myself. No one knows. (P20)

### Access to Strategies to Address Challenges Posed by the Impact of Long COVID on Employment

Two subthemes encompassed patients’ strategies to address the challenges posed by the impact of Long COVID on employment and their access to or barriers to accessing these strategies: (1) coping strategies and (2) employer disability benefits and workplace accommodations (see Appendix Table 4 for additional supporting quotations). Patients described strategies they used at work to help overcome the challenges of their Long COVID symptoms. For example, taking frequent rest periods was helpful to perform work tasks that were made difficult by fatigue, and note-taking/list-making was described as helpful to address challenges related to brain fog. As one participant described, “I do a daily report of what I do, and I email it to myself. … I have a notebook that I write in just so I can encapsulate what I did, because sometimes I forget.” (P2). Some patients reported that they learned these strategies from their healthcare providers, including occupational therapists, speech therapists, and cognitive therapists.

Patients also discussed interest in and use of employer disability benefits, like short-term or long-term disability, or workplace accommodations, such as working shifted hours or working remotely. Some patients described that options for disability or accommodation were brought to their attention by their healthcare team. Others reported interest in discussing this with their healthcare providers. While some patients reported supportive employers when requesting accommodations, many reported experiencing resistance or waning support over time when needing accommodations to adjust the type or timing of their work. One participant described their experience having initially received accommodation from their employer to work remotely, but eventually was pressured to return to an in-person office environment:

They basically said, ‘Oh, this role wasn’t meant to be virtual, and you have to be in office.’ Then they just kind of made up more and more things to make me feel uncomfortable. (P8)

## DISCUSSION

Our findings illuminate the experiences of patients with Long COVID who have been unable to return to their pre-COVID-19 levels of employment. The inability to return to work at their former capacity compounds the financial hardships that patients incur with the increased costs associated with their healthcare.^21^ The distress and diminished quality of life that result from the inability to meet financial demands may create a state of “financial toxicity”—encompassing the negative effects of the financial burden of healthcare costs—similar to what has been described for people with cancer.^22–25^ Furthermore, the loss of identity that accompanies an unwanted change in employment status may contribute to some of the mental health consequences observed in the Long COVID population.^26,27^ Understanding perceptions of identity in Long COVID may also draw from experiences in broader disability communities. For example, patients with Long COVID may or may not currently embrace “disability identity,” but acknowledgement of this identity may provide potential positive and empowering effects as well as a foundation to advocate for and support patients with Long COVID in community, workplace, and healthcare settings.^28,29^

Our study provides insight into perceptions of individuals with Long COVID at the intersection of employment, health, and well-being. Our work supplements and expands upon evidence in the literature that has also described the challenges that Long COVID presents to maintaining work-life balance,^10,16^ expressions of loss of professional identities,^10,12,15,16^ and barriers to accessing support, such as unemployment benefits or workplace accommodations.^13,14^ Studies have also called attention to the fluctuating nature of Long COVID that necessitates flexibility in working conditions.^15,16,30^ Multiple studies have investigated the roles of employer organizations, managers, and coworkers as facilitators or barriers to individuals maintaining employment while experiencing Long COVID.^9–12^ Fewer studies have recognized the role of healthcare providers in supporting individuals with Long COVID who face employment challenges.^16^

Our study adds to this literature by improving our understanding of the experiences of individuals with Long COVID receiving care at a post-COVID recovery clinic who face challenges with employment. Our findings include experiences described by our study participants that directly involved their healthcare providers, such as instances in which their healthcare providers made suggestions for changes in employment to prioritize recovery, provided support for seeking work accommodations, or offered ideas for coping mechanisms to help manage Long COVID symptoms that interfered with work tasks. The perspectives of our study participants also provided insight into more general aspects of their experiences navigating the management of their Long COVID illness along with the impact of their illness on their ability to work, including the toll on their work-life balance, financial situation, and mental health. Together, these findings help identify opportunities for healthcare providers to intervene, including providing recommendations for changes in employment, recognizing difficulties balancing work and healthcare appointments, assessing and addressing mental health repercussions of changes in work capacity, facilitating financial assistance, and offering support to pursue work accommodations. With these opportunities in mind, we suggest several areas in which healthcare providers can enhance support for patients with Long COVID who face challenges with employment.

First, it is critical that healthcare providers acknowledge Long COVID as a potentially disabling illness.^3,31,32^ Long COVID is recognized by the U.S. Department of Health and Human Services as a disability and is covered under the Family and Medical Leave Act and the Americans with Disabilities Act.^33^ While energy conservation techniques, including rest and pacing, are guideline-supported therapeutic interventions for Long COVID,^34–36^ providers should anticipate and address the impact of these recommendations on employment and well-being.

It is also important for healthcare providers to recognize the intertwined aspects of potential financial pressures caused by the impact of Long COVID on employment, the cost of Long COVID treatment, and patients’ health and well-being.^37,38^ For those patients who experience loss of income and/or high treatment costs, healthcare providers can offer connections to resources including opportunities for financial aid and assistance. Healthcare systems should consider the multidisciplinary support that roles such as providers, social workers, and financial counselors can offer to provide more comprehensive assistance through financial navigation programs.^39,40^ Established use of financial navigators for patients with other chronic conditions can provide inspiration for translation of these interventions to the Long COVID patient population.^39^

Additionally, it is imperative that providers acknowledge that changes in employment may negatively impact the mental health of individuals with Long COVID.^41–43^ There is an opportunity for healthcare providers to use established screening tools (e.g., Patient Health Questionnaire-9, General Anxiety Disorder-7) to assess the mental health of Long COVID patients. While there are standards of care for treating mental health conditions, guidance is needed to assist healthcare providers in screening for and treating these conditions among patients with Long COVID. Referral to rehabilitation psychology may be a valuable option to assist with documenting disability and making employment recommendations, while also addressing the mental health issues that may arise from navigating Long COVID and its impacts on work capacity.

Finally, healthcare providers have a critical role in providing supporting information to patients and employers through job accommodation requests. By documenting disability and supporting workplace accommodations, providers can favorably impact patients’ quality of life, illness trajectory, and access to health care. Providers can help guide patients through accommodation requests, where resources to help navigate these processes are often limited. Providers can also communicate with patients to determine reasonable duration of time off work, staged return, and workplace accommodations that allow continued employment during recovery. Inspiration may be sought in successful interventions that support employment among individuals with other chronic conditions.^44^ Due to lack of established practices to diagnose Long COVID, it is important to recognize the challenge of applying for work accommodation without objective diagnostic data.^45^ Accommodations can be requested based on documented functional limitations, rather than diagnostic testing,^45^ using questionnaires about symptoms and limitations for functional evaluation.^46,47^ Coordinating with organizational disability specialists to advise on documentation may increase provider confidence in handling accommodation requests. Referral to vocational rehabilitation counselors or physical, occupational, or speech therapists may be an additional strategy to provide support during changes to employment and transitions of return to work. These interactions may provide additional opportunities to evaluate functional capacity and review or suggest coping strategies for the management of Long COVID symptoms related to work, such as those mentioned by our study participants; these included utilizing lists and notes, reducing distractions in the workplace, and scheduling regular breaks.

### Limitations and Opportunities for Future Research

Our study has several limitations. First, our study population is restricted to those who had access to the post-COVID recovery clinic and may represent those who are particularly motivated to seek care for their Long COVID symptoms. Second, many of our participants were experiencing Long COVID symptoms for prolonged periods of time (e.g., greater than one year), suggesting a population enriched by those experiencing particularly severe Long COVID. Our findings may not be applicable to individuals who experience more mild cases of Long COVID. Third, we did not restrict our study population to those who experienced work-related limitations due to Long COVID. Our findings therefore include a breadth of perspectives on the extent to which Long COVID impacted individuals’ abilities to work. Fourth, our recruitment was selective of individuals who self-reported doing well in daily life prior to COVID-19. We did not screen for pre-existing conditions that might impact employment, disability, or mental health. Fifth, we did not collect information about our study participants’ race/ethnicity to compare unique experiences according to this demographic information. Finally, we acknowledge that participation in our study was voluntary, and therefore, those patients who refused to participate may have perspectives that are different from those expressed by patients who agreed to be interviewed.

Opportunities for future work include expanding our understanding of the experiences of individuals with milder cases of Long COVID and individuals with pre-existing conditions or comorbidities that can further exacerbate the negative impacts of Long COVID.^48^ Future research should also explore the perspectives of historically marginalized populations^49^ and include longitudinal analyses aimed at understanding the long-term consequences of Long COVID on employment, financial stability, and mental health.

## CONCLUSIONS

This study expands our knowledge about the experiences of Long COVID patients as they navigate challenges at the intersection of health and employment. These findings highlight the need to fill significant gaps in support systems for individuals with Long COVID by connecting them to resources for financial assistance, mental health treatment, and workplace accommodation requests. Concerted efforts are needed from healthcare systems to help patients manage their complex Long COVID symptoms while facing challenges with employment.

## Supplementary Information

Below is the link to the electronic supplementary material.

Supplementary file1 (DOCX 38 KB)

## Data Availability

Data will not be made publicly available due to concerns for participant privacy.
