# Supplementary material for: The Impact of Long COVID on Employment and Well-Being: A Qualitative Study of Patient Perspectives
Source: J Gen Intern Med. 2024 Oct 8;40(5):1070–7. doi: 10.1007/s11606-024-09062-5 (PMC11968618; doi:10.1007/s11606-024-09062-5)
Supplement: Supplementary file 1 — Supplementary file1 (DOCX 38 KB) [file 11606_2024_9062_MOESM1_ESM.docx]

**APPENDICES**

**Table 1. Impact of Long COVID on the ability to work**

| **Impact** | **Patient Quotations** |
| --- | --- |
| Interference of Long COVID symptoms with work responsibilities | The shortness of breath was really, really was hard on me. At work, my job is to walk around … and many times, I would just have to stop, find a place to sit down. I would do my breathing, and rest for a little bit, and then get up and go again. Mostly I learned to listen to my body and understand that my body is in charge, not my brain. My heart and my brain want to do it, but my body can't. (P21)  I wasn't able to carry on the work schedule the way that I had. Honestly, probably at that point I was only good for, you know, a couple hours a day, and that would wear me out. And I couldn't problem-solve, and things just kind of kept, they didn't get better. (P16) |
| Impact of Long COVID on work-life balance | I like to be engaged with the world and like doing things, but now I just don't have the energy to live that kind of life anymore. … I have to get through my workday, which is a struggle. And then I go home, and I’m wrecked. (P1)  I tried to go back to work, and I worked two half days, and I slept for almost 20 hours after that. I just, it just completely, completely wiped me out. I'm talking about a deep, deep, sleep for 20 hours. (P7) |

**Table 2. Financial consequences from the impact of Long COVID on employment**

| **Financial Consequence** | **Patient Quotations** |
| --- | --- |
| Loss of income | I cut back on my hours. … 3 hours a day I was losing, or three hours a week I should say. So, three hours a week. My check was that much shorter. (P21)  I usually work overtime. I’m supposed to work three 12-hour days a week. And most pay periods I work at least one extra day. So, I missed out on extra pay. (P5) |
| Changes in insurance | I got the job, the other job at [company name], for the insurance literally. And so, I was lucky enough, I guess to get COVID while I was on that insurance. I had, in two months, paid the entire deductible, which was painful, max out-of-pocket. By the end of [month], I completely paid my max out-of-pocket for the entire plan, but then got laid off. So, then I was without, so I was on Medicaid. Which, okay, then they paid for stuff. And now I'm starting over again. I'm with a new job. Medicaid's like, we're secondary now. And now I'm starting over, paying yet again. (P3)  I left my medical job because all that stuff that was happening there was not good for my mental health. So, I had to get on my husband's insurance. And so that restarted my deductible. So that part didn't help either. (P8) |

**Table 3. Emotional repercussions from the impact of Long COVID on employment**

| **Emotional Repercussion** | **Patient Quotations** |
| --- | --- |
| Feelings of loss | Trying to be optimistic about getting better, then trying to be a little bit more realistic about what my future looks like. Trying to take the steps to put certain strategies in place so that I can function at work. Recognizing what my struggles are. Trying to think about maybe, what am I going to do professionally? I thought I was going to have to leave my job. And I still like struggle so much that I kind of have to think about, okay, what does the next stage of my life look like? (P1)  Yeah, I can't work anymore. That's a huge thing for me. That's a huge stigma because it kind of makes our lives more difficult financially … and my poor husband has to support us, and I feel guilty. (P6) |
| Fear of exposure, judgement, stigma, and/or retaliation | From a work perspective, being discriminated against, laid off, having to tell people up front that I have it when I'm applying, like things weren't crazy enough out there. Being older … and telling them I have [Long] COVID. (P3)  Especially when people that, you know, matters to you, like your family, you know, like my dad. You know, he's older, he's a hard worker and I know that he has lost a lot of respect for me because I don't work anymore. That's hard. … Primarily the fatigue that makes me look like I'm lazy and I don't think people understand that it's just, I don't have a whole lot of control over it. I'm not just, you know, I'm not lying in bed, just because I'm lazy. I’m lying in bed, because my body doesn't have the strength to do what I want to do. (P7) |

**Table 4. Access to strategies to address challenges posed by the impact of Long COVID on employment**

| **Strategy** | **Patient Quotations** |
| --- | --- |
| Coping strategies | And my job was such that as long as I get the work done, it didn't matter. So, I could go lay down and come back. I could work on weekends a little bit and do whatever I needed to, to put in all the hours to get the work done. (P3)  I'm able to concentrate more when I'm not in an office where people start conversations that draw my attention away from my work or want to come up and interrupt my day with something they just want to talk about that’s unrelated. (P17) |
| Employer disability benefits and workplace accommodations | I tried to get disability and they denied me twice. And I didn't do it solely on the basis of I had COVID-19. And I don’t know if I'm a long-term [Long COVID] or not. … I don't know at all. I never heard that phrase until my cardiologist said it. And they still denied me because they don't believe any of these symptoms. (P6)  My current boss doesn't always understand that I am not able to put in all the time that he would like me to. And I have struggled to stay within the realms of the ADA [Americans With Disabilities Act] accommodations I've been given. … There's a lot of pressure to where I'm like, I wasn't feeling well. ‘Oh well, you can make that time back up.’ I'm like, no, it's part of my accommodation. (P14) |
